# Intra‐Abdominal Myxoid Leiomyosarcoma With PLAG1 Gene Rearrangement Following Prior Laparoscopic Myomectomy: A Case Report and Literature Review

**DOI:** 10.1002/ccr3.73539

**Published:** 2026-09-16

**Authors:** Cheng‐En Hsieh, Po‐Hsuan Wu, Chang‐Wei Su, Yu‐Chun Ma, Hsuan‐Ying Huang

**Affiliations:** ^1^ Kaohsiung Medical University Kaohsiung City Taiwan; ^2^ Kaohsiung Medical University Chung‐Ho Memorial Hospital Kaohsiung City Taiwan; ^3^ Kaohsiung Medical University Gangshan Hospital Kaohsiung City Taiwan; ^4^ Kaohsiung Chang Gung Memorial Hospital and Chang Gung University College of Medicine Kaohsiung City Taiwan

**Keywords:** gene rearrangement, immunohistochemistry, laparoscopic myomectomy, PLAG1 gene, uterine leiomyoma, uterine leiomyosarcoma

## Abstract

PLAG1 immunohistochemistry and FISH were essential for diagnosis of extra‐uterine myxoid leiomyosarcoma and should be considered in myxoid intra‐abdominal tumors, especially in patients with previous uterine surgery.

## 1 Introduction

Myxoid leiomyosarcoma (mLMS) is a rare morphological variant of leiomyosarcoma distinguished by its abundant myxoid stroma, which separates neoplastic smooth muscle cells and imparts a deceptively bland histological appearance. While the uterus is the most common primary site, extra‐uterine occurrences involving the retroperitoneum, intra‐abdominal cavity, and soft tissues are exceedingly rare [1]. Recent population‐based data confirm its extreme rarity and identify elderly females as the demographic most affected, with the uterus representing the predominant primary site among myxosarcoma subtypes [1].

The prognosis of mLMS is a subject of ongoing debate. Some series report a highly aggressive clinical course with a 5‐year overall survival as low as 11.1%, driven by a high propensity for local recurrence and distant metastasis [2]. In contrast, a Norwegian population‐based study reported a 5‐year survival approaching 73%, slightly more favorable than conventional leiomyosarcoma, suggesting that the clinical heterogeneity of mLMS may reflect underlying molecular diversity [2]. This prognostic variability underscores the importance of accurate molecular characterization.

Diagnostically, mLMS presents formidable challenges due to its morphological and radiological overlap with a spectrum of benign and malignant myxoid lesions, including myxoid leiomyoma, aggressive angiomyxoma, and low‐grade endometrial stromal sarcoma [3]. Furthermore, recent molecular studies have identified PLAG1 gene rearrangement as a recurrent and diagnostically significant alteration in a subset of mLMS, providing a critical tool for distinguishing this entity from its morphological mimics [4, 5]. Currently, while standardized treatment guidelines remain elusive, complete surgical excision with negative margins represents the cornerstone of curative‐intent management [6].

## 2 Case History, Examination, Differential Diagnosis

A 39‐year‐old female with a history of laparoscopic myomectomy for a 13‐cm submucosal uterine myoma 2 years prior presented with a 1‐month history of intermittent left‐sided abdominal dull pain aggravated by palpation, accompanied by a recent unintentional 3‐kg weight loss. Physical examination revealed mild left abdominal tenderness without peritoneal signs. Laboratory investigations including complete blood count, biochemistry, and tumor markers were unremarkable.

**FIGURE 1:**
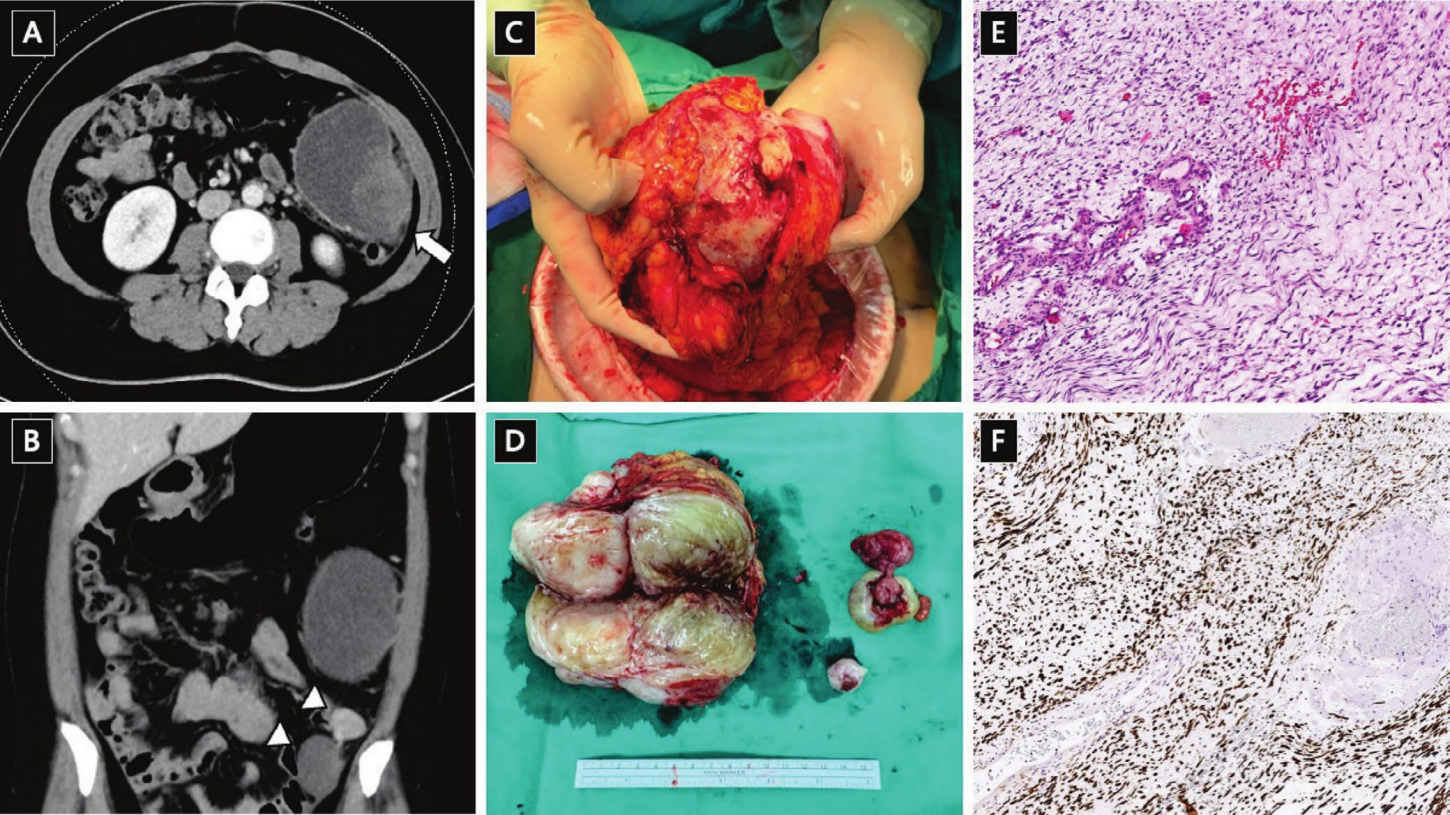
Radiologic, operative, gross, and histopathologic features of intra‐abdominal myxoid leiomyosarcoma with PLAG1 gene rearrangement. (A) Axial contrast‐enhanced CT scan demonstrating a large, poorly enhanced, heterogeneous soft tissue mass in the left peritoneal space (white arrow), measuring 11.8 cm. (B) Coronal CT image revealing a separate complex cystic‐solid lesion in the left lower abdomen (white arrowheads), measuring 5.8 cm, consistent with a second tumor deposit. (C) Intraoperative photograph demonstrating the large lobulated intra‐abdominal tumor during en bloc resection. (D) Gross pathological specimen showing a multilobulated mass with a gelatinous myxoid cut surface. A small satellite nodule is identified adjacent to the main tumor. (E) Hematoxylin and eosin staining demonstrating spindle cell proliferation within abundant myxoid stroma, with mild nuclear atypia (original magnification ×100). (F) Immunohistochemical staining for Desmin demonstrating strong and diffuse cytoplasmic positivity in tumor cells, confirming myogenic lineage despite the myxoid background (original magnification ×100).

Contrast‐enhanced abdominal CT revealed a large, poorly enhanced, heterogeneous soft tissue mass in the left peritoneal space (11.8 cm) (Figure 1A, arrow) and a separate complex cystic‐solid lesion in the left lower abdomen (5.8 cm) (Figure 1B, arrowheads), raising the possibility of a primary intra‐abdominal neoplasm with satellite lesion or multifocal disease. A CT‐guided percutaneous core needle biopsy suggested an atypical myxoid neoplasm without definitive classification.

## 3 Treatment and Investigations

For concurrent definitive diagnosis and therapeutic resection, intra‐abdominal tumor excision was subsequently performed (Figure 1C). Grossly, the resected specimen revealed a multilobulated mass with a gelatinous myxoid cut surface, measuring 11.8 cm at its greatest dimension, with a small adjacent satellite nodule (Figure 1D).

Histopathological examination demonstrated spindle cell proliferation with mild nuclear atypia and high mitotic activity (the mitotic rate is 26 mitoses/10 high power fields), embedded within an abundant myxoid stroma (Figure 1E). Immunohistochemically, tumor cells showed strong and diffuse Desmin positivity (Figure 1F), indicating myogenic lineage, but were negative for SMA, h‐Caldesmon, and SMMHC, consistent with the incomplete smooth muscle differentiation profile characteristic of mLMS [2]. The tumor additionally co‐expressed ER, PR, CD10, CD34, and Cyclin D1. Strong and diffuse nuclear PLAG1 protein overexpression was detected by immunohistochemistry, prompting molecular workup. MDM2 protein overexpression was also identified; however, subsequent FISH confirmed a PLAG1 break‐apart signal consistent with PLAG1 gene rearrangement, while MDM2 gene amplification was absent, effectively excluding well‐differentiated and dedifferentiated liposarcoma. Integrating the histomorphological pattern, immunophenotype, and molecular findings, a definitive diagnosis of mLMS harboring PLAG1 gene rearrangement was rendered.

## 4 Results (Outcome and Follow‐Up)

The patient recovered smoothly postoperatively, experiencing only transient abdominal distention, and was discharged on postoperative day 10. At the 6‐month outpatient follow‐up, she remained completely asymptomatic with no clinical or radiological evidence of tumor recurrence.

## 5 Discussion and Conclusions

mLMS is a rare and diagnostically challenging malignant smooth muscle neoplasm characterized by spindled tumor cells embedded within an abundant myxoid matrix. Although it predominantly affects the uterus, extra‐uterine manifestations involving the soft tissue, retroperitoneum, and intra‐abdominal cavity have been documented, albeit infrequently [1]. The extreme rarity of extra‐uterine mLMS means that its clinicopathological behavior remains poorly characterized, and established management protocols are largely extrapolated from data on uterine leiomyosarcoma.

The prognosis of mLMS is subject to considerable debate in the literature. Parra‐Herran et al. reported a 5‐year overall survival of only 11.1% in a series with a high proportion of tumors meeting conventional LMS diagnostic criteria, suggesting aggressive behavior [2]. Conversely, a population‐based Norwegian study found a 5‐year survival of 73%, and Burch and Tavassoli reported recurrence in only 5 of 12 cases over 3–9 years of follow‐up [2]. This discrepancy likely reflects heterogeneity in diagnostic criteria and underlying molecular subtypes. The large‐scale molecular profiling study by Dundr et al. [4] further underscored the molecular diversity within uterine smooth muscle tumors, emphasizing that molecular classification refines both diagnosis and prognosis. As such, the prognostic significance of specific alterations such as PLAG1 rearrangement warrants dedicated prospective investigation.

The clinical presentation of extra‐uterine mLMS is nonspecific, with symptoms primarily attributable to tumor mass effect or local invasion. Radiologically, the myxoid matrix imparts low‐attenuation or heterogeneous signal characteristics that frequently mimic benign myxoid lesions, cystic structures, or other soft tissue tumors, rendering imaging insufficient for definitive characterization [3]. In the present case, two spatially separate lesions identified on CT raised the diagnostic possibility of multicentric disease or peritoneal dissemination, underscoring the need for tissue confirmation in all such cases.

Immunohistochemistry plays a pivotal role in the diagnosis of mLMS, yet its interpretation requires awareness of the variable expression of smooth muscle markers in this entity. Unlike conventional leiomyosarcoma, mLMS frequently demonstrates reduced or absent reactivity for SMA and h‐Caldesmon, while desmin positivity is retained in most cases, reflecting an incomplete myogenic differentiation profile [2]. In the present case, strong desmin positivity with concurrent negativity for SMA, h‐Caldesmon, and SMMHC exemplified this characteristic pattern. The additional co‐expression of CD10, Cyclin D1, ER, and PR prompted consideration of endometrial stromal sarcoma; however, the retention of desmin positivity, the morphological context, and subsequent molecular results argued against this diagnosis [4, 5].

PLAG1 gene rearrangement has been identified in approximately 25% of uterine mLMS cases and represents a highly specific molecular biomarker for this entity, distinguishing it from ZC3H7B‐BCOR high‐grade endometrial stromal sarcoma and myxoid inflammatory myofibroblastic tumor [5]. Strong and diffuse nuclear PLAG1 immunoexpression, as observed in the present case, reliably identifies tumors harboring PLAG1 gene rearrangement and serves as a practical triage tool prior to confirmatory FISH [5]. The absence of MDM2 gene amplification by FISH, despite MDM2 protein overexpression on immunohistochemistry, effectively excluded well‐differentiated and dedifferentiated liposarcoma, which can morphologically overlap with myxoid lesions.

Of particular clinical significance in this case is the prior laparoscopic myomectomy performed 2 years before the current presentation. Devaud et al. [6] emphasized that myomectomy is strictly indicated for benign leiomyomas, and that inadvertent surgery on an occult leiomyosarcoma carries the risk of intraperitoneal tumor dissemination even in the absence of morcellation, as tumor cells have been detected in peritoneal fluid following myomectomy alone. The present multilesional intraperitoneal distribution—two spatially separate masses with an adjacent satellite nodule—raises strong suspicion that iatrogenic peritoneal seeding secondary to the prior myomectomy may have contributed to the current tumor distribution. This hypothesis has important implications: any leiomyosarcoma diagnosis arising after a prior myomectomy warrants complete surgical re‐evaluation, including hysterectomy, and close surveillance for intraperitoneal disease [6]. Moreover, this case reinforces the critical need for accurate preoperative differentiation between benign leiomyoma and leiomyosarcoma, particularly in younger patients with large or atypical uterine masses.

Regarding treatment, complete surgical resection with negative margins remains the cornerstone of curative therapy for localized mLMS [6]. Given the high rates of metastatic failure following surgical resection, systemic therapy is an important consideration for advanced disease. Doxorubicin has long been the standard first‐line agent for metastatic leiomyosarcoma; however, the LMS‐04 Phase III randomized controlled trial demonstrated that doxorubicin combined with trabectedin significantly improved progression‐free survival compared with doxorubicin monotherapy as first‐line therapy for metastatic or unresectable leiomyosarcoma [7]. These findings represent an important advance in systemic management and should inform treatment planning for patients with unresectable or metastatic mLMS.

In conclusion, this case illustrates the diagnostic and therapeutic complexity of extra‐uterine mLMS with PLAG1 gene rearrangement in the context of prior uterine surgery. The combination of an incomplete smooth muscle immunophenotype, strong diffuse nuclear PLAG1 expression, and confirmatory FISH was instrumental in establishing a definitive molecular diagnosis. The history of prior laparoscopic myomectomy adds a critical clinical dimension, raising the possibility of iatrogenic peritoneal seeding and emphasizing the importance of preoperative risk stratification in patients undergoing uterine surgery. This case emphasizes that comprehensive molecular workup—including PLAG1 immunohistochemistry and FISH—should be considered in all myxoid intra‐abdominal tumors, particularly in patients with a prior history of uterine surgery.

## Author Contributions

**Cheng‐En Hsieh:** writing – original draft. **Po‐Hsuan Wu:** conceptualization, writing – review and editing, supervision. **Chang‐Wei Su:** supervision, writing – review and editing. **Yu‐Chun Ma:** resources. **Hsuan‐Ying Huang:** resources.

## Funding

The authors have nothing to report.

## Ethics Statement

All procedures performed in studies involving human participants were in accordance with the ethical standards of the Institutional and/or National Research Committee and with the 1964 Helsinki Declaration and its later amendments or comparable ethical standards.

## Consent

Written informed consent was provided for the publication of this case report, consistent with the journal's requirements regarding patient consent.

## Conflicts of Interest

The authors declare no conflicts of interest.

## Data Availability

The data supporting the findings of this study are available from the corresponding author upon reasonable request.
